# Change in model for end-stage liver disease score at two weeks, as an indicator of mortality or liver transplantation at 60 days in acute-on-chronic liver failure

**DOI:** 10.1093/gastro/gou075

**Published:** 2014-11-11

**Authors:** Rajneesh Kumar, Thinesh Lee Krishnamoorthy, Hiang Keat Tan, Hock Foong Lui, Wan Cheng Chow

**Affiliations:** Department of Gastroenterology and Hepatology, Singapore General Hospital, Singapore

**Keywords:** acute-on-chronic liver failure, model for end-stage liver disease (MELD) score, liver transplantation, mortality

## Abstract

**Background:** Acute-on-chronic liver failure (ACLF) is characterised by a sudden deterioration of underlying chronic liver disease, resulting in increased rates of mortality and liver transplantation. Early prognostication can benefit optimal allocation of resources.

**Methods:** ACLF was defined as per the disease criteria of the Asian Pacific Association for the Study of the Liver. Inpatient discharge summaries from between January 2001 and April 2013 were reviewed. The primary outcome was mortality or liver transplantation within 60 days from onset of ACLF. Absolute ‘model for end-stage liver disease' (MELD) score and change in MELD at Weeks 1, 2 and 4 were reviewed in order to identify the earliest point for prediction of mortality or liver transplantation.

**Results:** Clinical data were collected on 53 subjects who fulfilled the inclusion and exclusion criteria. At 60 days from presentation, 20 patients (37.7%) died and 4 (7.5%) underwent liver transplantation. Increased MELD of ≥2 after 2 weeks was 75.0% sensitive and 75.9% specific for predicting mortality or liver transplantation. If the MELD score did not increase at 2 weeks, predictive chance of survival was 93.8% over the next 60 days. MELD change at 1 week showed poor sensitivity and specificity. Change at 4 weeks was too late for intervention.

**Conclusion:** Change in MELD score at 2 weeks provides an early opportunity for prognostication in ACLF. A MELD score that does not deteriorate by Week 2 would predict 93.8% chance of survival for the next 60 days. This finding warrants further validation in larger cohort studies.

## Introduction

Acute-on-chronic liver failure (ACLF) is an increasingly recognised clinical entity, characterised by the sudden worsening of underlying chronic liver disease with development of jaundice, ascites, hepatic encephalopathy and/or a coagulopathy, which results in increased mortality and need for liver transplantation [1]. Whereas the predominant aetiology in the West is alcohol and drugs, infectious causes are more common in the East [1]. This condition can develop in both cirrhotic and non-cirrhotic patients with a background of chronic liver disease. Early recognition of this condition is important for prognostic purposes and to start aggressive treatment of patients, for whom only a narrow therapeutic window of opportunity may exist.

Although the clinical condition is being increasingly recognised, an East–West difference remains in respect of the actual parameters that make up the definition of this condition. The Asian Pacific Association for the Study of the Liver has produced a working definition of this entity [2].

ACLF is associated with a high mortality of 50–90% [3]. Whilst the King’s College criteria are used in prognostication for acute liver failure [4], and the ‘model for end-stage liver disease' (MELD) score is used in chronic liver disease for both prognostication and priority listing for liver transplantation [5], no universally accepted prognostic model exists for ACLF.

In a resource-limited setting like Singapore, where cadaveric livers are not readily available, prioritizing those patients most in need of life-saving liver transplantation is essential. With this study, we attempted to describe the aetiology of presentations to our unit, as well as to assess the utility of the MELD score in early prognostication for ACLF.

## Patients and methods

This study was approved by the Institutional Review Board of SingHealth Services, which includes Singapore General Hospital, a tertiary-level centre for chronic liver disease and liver transplantation. Waiver of informed patient consent was approved by the Institutional Review Board. A retrospective review of case notes was conducted on all patients admitted to this institution for decompensated liver disease between January 2001 and April 2013. Clinical and biochemical data was scrutinized to determine eligibility for this study. Data collection included patient demographics, clinical parameters, laboratory values, aetiology of both acute insult and underlying chronic liver disease, and time to mortality and/or liver transplantation.

ACLF was defined by the APASL (Asia pacific association for study of Liver) criteria, i.e. serum bilirubin >85μmol/L (5mg/dL), international normalized ratio (INR) >1.5, and ascites and/or hepatic encephalopathy (HE) in a patient with previously diagnosed or undiagnosed chronic liver disease [2]. Patients were excluded if they were under 21 years old or if they had bland cholestasis as the underlying liver disease. Definition of cirrhosis was based on liver biopsy or standardized clinical, laboratory, ultrasonographic and endoscopic criteria. HE was defined by the presence of Grade 2 or higher encephalopathy, based on the West Haven criteria [6].

The primary endpoint of this study was mortality or liver transplantation at 60 days. Additional data on mortality were collected up to 1 year from presentation. For the purposes of this study, patients were divided into those who died or underwent liver transplantation within 60 days (Group 1) and those who survived past 60 days without liver transplantation (Group 2). We collected information on the underlying chronic liver disease of patients admitted for ACLF and aetiologies of the acute insult, and calculated the MELD score at 1, 2 and 4 weeks following diagnosis of ACLF.

### Statistical analysis

Results were analysed using SPSS version 21.0 (IBM Corp, Armonk NY). Continuous variables were expressed as mean ± standard deviation (SD). Categorical variables are expressed as actual numbers and their percentages. Analysis of variance was used to compare the differences in variables between the two groups. Group comparisons of categorical variables were analysed with the χ^2^ test. A *P-*value of <0.05 was considered statistically significant. Kaplan-Meier curves were used to illustrate survival data and log-rank tests were used to test for statistically significant survival.

## Results

### Demographic and clinical characteristics

Inpatient summaries from January 2001 to April 2013 were reviewed and data were available for 53 subjects at the time of reporting. The mean age of patients was 55.9 ± 10.7 years, with 73.6% being male. Baseline demographics and clinical parameters are shown in Table 1. The racial mix was Chinese 77.4%, Indian 20.8% and other races 1.9%. The aetiology of the chronic liver disease was dominated by chronic hepatitis B (66.0%) and alcoholic liver disease (24.5%). The acute insult was a hepatitis B-related event in 52.8% of cases. Alcohol and drugs accounted for the other major inciting events in 18.9% and 9.5%, respectively.

**Table 1.** Demographic and clinical characteristics

Variable	Total (*n = *53)	Group 1. Died or transplanted	Group 2. Survived	*P-*value
		(*n = *24)	(*n = *29)	
Gender: *n* (%)				0.99
Male	39 (73.6)	15 (62.5)	24 (82.8)	
Female	14 (26.4)	9 (37.5)	5 (17.2)	
Race: *n* (%)				
Chinese	41 (77.4)	21 (87.5)	20 (69.0)	
Indian	11 (20.8)	2 (8.3)	9 (31.0)	
Others	1 (1.9)	1 (4.2)	0 (0)	
Age: years	55.9 ± 10.7	56.9 ± 11.2	55.1 ± 10.3	0.54
Aetiology of liver disease: *n* (%)				**0.03**
Chronic hepatitis B	35 (66.0)	20 (83.4)	15 (51.7)	
Alcohol	13 (24.5)	2 (8.3)	11 (38.0)	
Others	5 (9.5)	2 (8.3)	3 (10.3)	
Aetiology of acute insult: *n* (%)				**0.37**
Hepatitis B flare	28 (52.8)	18 (75)	10 (34.4)	
Alcoholic hepatitis	10 (18.9)	2 (8.3)	8 (27.5)	
Drug-induced liver injury	5 (9.4)	2 (8.3)	3 (10.3)	
Others	10 (18.9)	2 (8.3)	8 (27.5)	
Cirrhosis present: *n* (%)	35/53 (66)	13 (54.2)	22 (75.8)	0.15
Encephalopathy present: *n* (%)	16 (30.2)	12 (50)	4 (13.8)	0.004
MELD score at:				
presentation	22.8 ± 4.7	24.5 ± 5.5	21.4 ± 3.3	0.015
Week 1	23.4 ± 5.4	26.2 ± 5.8	21.1 ± 3.7	<0.001
Week 2	26.4 ± 10.4	33.9 ± 9.9	20.2 ± 5.6	<0.001

MELD = model for end-stage liver disease

### Clinical outcomes

At 60 days following presentation, 20 patients (37.7%) died and 4 (7.5%) underwent liver transplantation. Of the four liver transplant patients, three are doing well at the time of reporting, while one succumbed to sepsis 6 months after transplantation. Mortality was higher in patients with hepatic encephalopathy (*P = *0.004) with an odds ratio (OR) of 6.25 (95% CI 1.7–23.5). Interestingly, cirrhosis at baseline was not associated with a higher incidence of mortality (*P = *0.15) (Table 1).

### Model for end-stage liver disease score

Of those patients with a peak MELD score >29 within the study period, 79.3% either died or underwent liver transplantation. Although 95.8% of patients in Group 1 reached a peak MELD score of >29, 66.7% actually reached this value at 2 weeks (Table 2). Higher MELD scores were observed in Group 1 at presentation but there was a large overlap with Group 2, demonstrated by a wide standard deviation. As time progressed, the absolute MELD scores between the two groups became statistically significant but there was still a large overlap (Table 1). The four patients undergoing liver transplantation had a mean MELD score of 36.7 that would conventionally have equated to a mortality of more than 50% at 90 days.

**Table 2.** MELD score and patient distribution

MELD score	<23	24–29	>29
Peak MELD	0/15	1/9	23/29
MELD at 2 weeks	4/24	4/10	16/19

MELD = model for end-stage liver disease

Numerator represents the number of patients who died or needed liver transplant.

Denominator represents the total number of patients with that MELD score.

### Change in model for end-stage liver disease score

**Figure 1.**
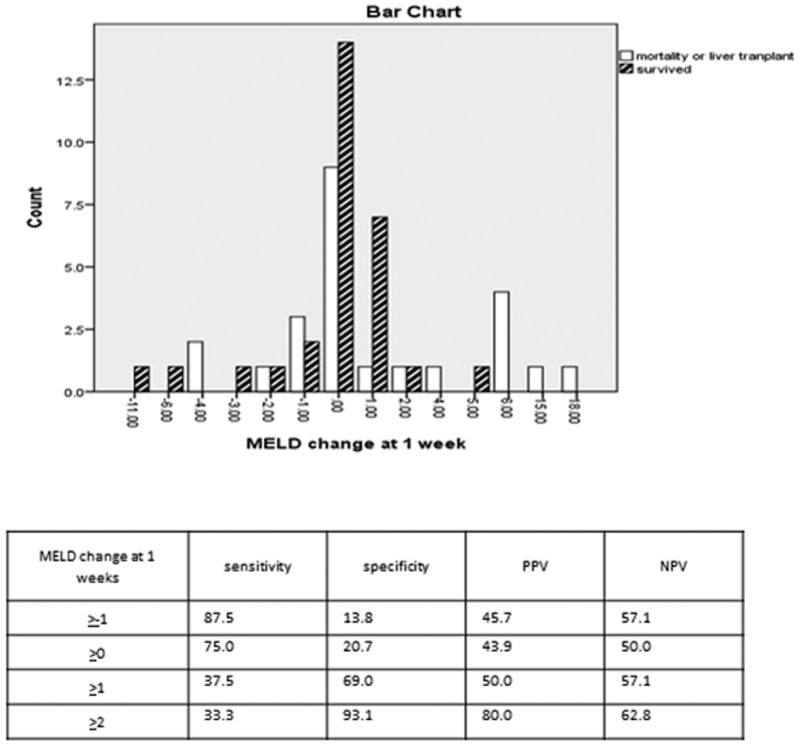
Model for end-stage liver disease change at 1 week. MELD change = MELD at 1 week minus MELD at presentation. PPV = positive predictive value; NPV = negative predictive value

If MELD score remained the same or increased at 1 week, it yielded a sensitivity of 75.0% (95% CI 53.3–90.2) but poor specificity of 20.7% (95% CI 8.1–39.7) for predicting mortality or liver transplant (Figure 1). Although the MELD scores between the two groups were statistically significant at 1 week, standard deviation was deemed excessively wide.

**Figure 2.**
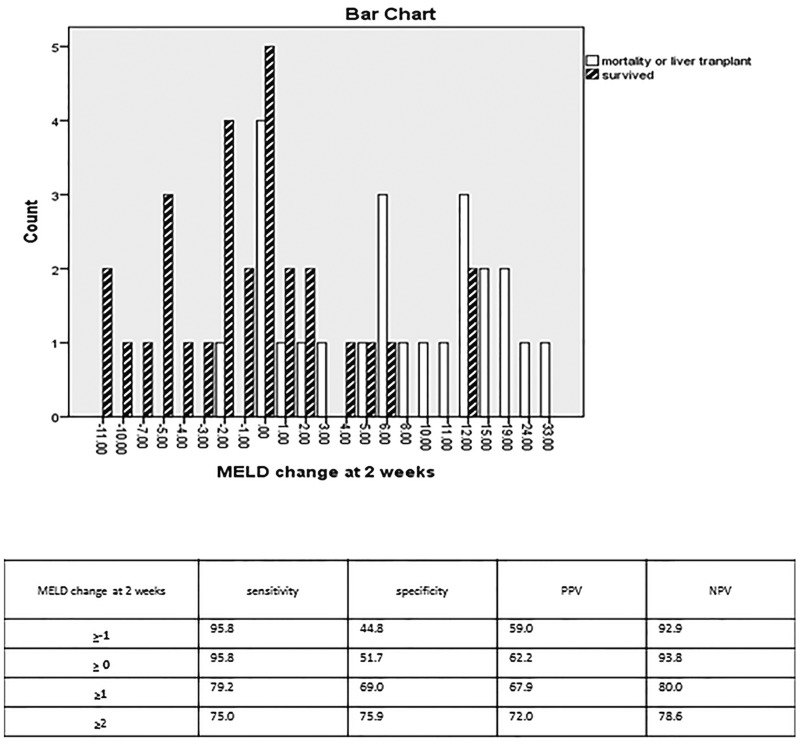
Model for end-stage liver disease change at 2 weeks. MELD change = MELD at 2 weeks minus MELD at presentation. PPV = positive predictive value; NPV = negative predictive value

At the end of Week 2 we found that the change in MELD score between the two groups became even more significant (Figure 2). At this point, if the MELD score remained the same or increased, it yielded a sensitivity of 95.8% (95% CI 78.8–99.3) and specificity of 51.7% (95% CI 32.5–70.5) for predicting mortality or need for liver transplantation, with an excellent negative predictive value of 93.8% (95% CI 69.7–99.0). Correspondingly, an increase in the MELD score of ≥1 at 2 weeks was 79.2% sensitive (95% CI 57.8–92.8) and 69.0% specific (95% CI 49.2–84.7) for predicting mortality or liver transplantation.

Additionally we noted that the greater the changes in the MELD score, the higher the specificity in predicting mortality or transplant. If the MELD score increased by ≥2 at 2 weeks, the sensitivity and specificity were 75.0% (95% CI 53.3–90.2) and 75.9% (95% CI 56.5–89.7) with positive and negative predictive values of 72.0% and 78.6%, respectively.

**Figure 3.**
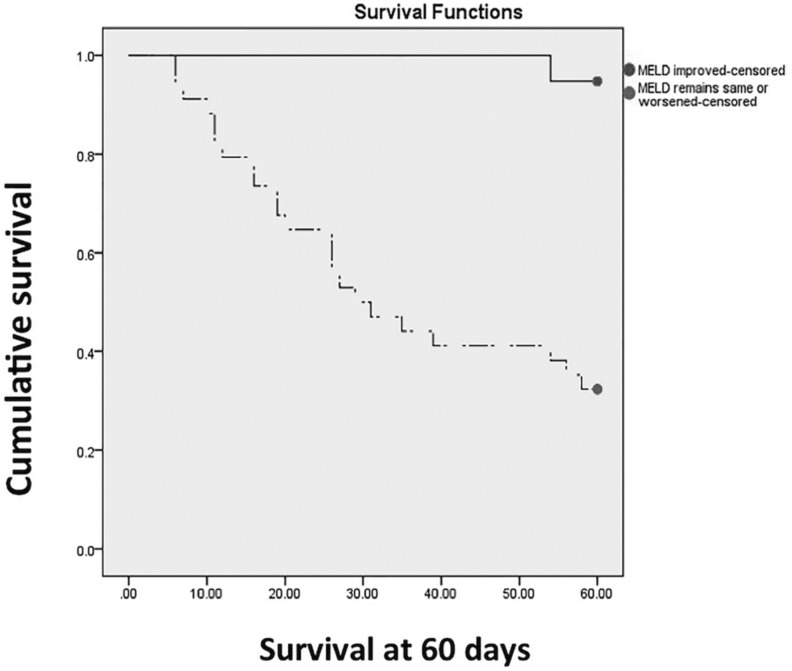
Improvement in MELD at 2 weeks. Straight line = MELD improved at 2 weeks. Broken line = MELD worsened at 2 weeks or remained the same. Log rank test *P < *0.001

We used a Kaplan-Meier survival model to demonstrate that a MELD score that improves at 2 weeks from onset of ACLF was strongly prognostic for survival at 60 days (Figure 3). If the MELD score did not increase at 2 weeks, predictive chance of survival was 93.8% over the next 60 days.

In a subgroup analysis of patients with chronic Hepatitis B infection, we assessed to see if a change in MELD score was valid and found it equally significant with log rank significance of 0.003. (Data not shown.)

## Discussion

Prognostic markers such as age, hepatic encephalopathy, MELD score, total bilirubin and INR have been hampered by heterogeneity of data, as demonstrated in a meta-analysis by Wlodzimirow and colleagues [7]. Earlier studies assessed mortality at 90 days, but we decided to assess mortality/transplantation at 60 days, given the rapid deterioration and inpatient mortality [1, 3].

The King’s College criteria have been widely used in acute liver failure (ALF) settings to select patients for transplantation [4], but no studies to date have investigated the their application in ACLF. Other attempts to prognosticate for ACLF have included the use of the MELD, sequential organ failure assessment (SOFA) and Acute Physiology and Chronic Health evaluation II (APACHE II) scores. Garg *et al.* found that, at baseline, all three scores are predictors for mortality [8], and follow-up studies suggested that hepatic venous pressure gradients and systemic haemodynamic changes could also predict mortality [9]. These studies focussed on endpoints of mortality and predictors, without taking into account the rapid dynamic changes in ACLF.

The SOFA score has been proposed to be prognostic for ACLF but the variables needed to calculate the SOFA scores were not reliably documented in the case notes used in this study and thus were omitted.

The MELD score was initially designed to assess mortality in patients after transjugular intrahepatic portosystemic shunt (TIPS) and was subsequently modified for prioritizing patients with decompensated chronic liver disease awaiting liver transplantation [10, 11]. A MELD score >29 is conventionally associated with mortality of 52% at 3 months [11]. However, the same score in our cohort was associated with a much higher mortality/transplantation rate of 79% at only 2 months, suggesting that the use of MELD score alone underestimates the severity of ACLF and the increased short-term mortality. Additionally, in our series, patients with poor outcomes had a mean transplant-free survival of less than four weeks, suggesting that, by the time their MELD scores exceed 29, insufficient time remained to prepare for liver transplantation. Along the same lines as our findings, Xia *et al.* reported that MELD scores had poorer prognostic value and went on to identify five factors associated with survival: MELD score, age, encephalopathy, trigycerides and platelet level [12]. Similarly, Yan *et al.* reported higher short-term mortality in patients with ACLF and the authors proposed integration of hepatic encephalopathy, age and MELD scores in patients with hepatitis B-related ACLF [13].

A recent study by Zheng *et al.* described dynamic changes in MELD scores for patients with acute-on-chronic hepatitis B liver failure, and its impact on prognosis [14]. The study found that a change in MELD score at 4 weeks was effective in determining the outcome. In our study however, an interval of 2 weeks was sufficient to demonstrate an increased mortality or need for liver transplant. This could potentially give the attending physician earlier prognostic data following conventional treatment.

Surprisingly, the presence of cirrhosis did not portend a poorer prognosis once the diagnosis of ACLF had been established. In our subjects, most of the cirrhotic patients had baseline Child-Turcotte-Pugh’s A or B liver cirrhosis before developing ACLF. However, clinical data relating to the time prior to presentation with ACLF was available for only 35 patients: for some, ACLF represented their first presentation. Hepatic encephalopathy was associated with increased mortality and this has been observed in previous studies in patients with ACLF [15]. The presence of Grade 2 hepatic encephalopathy was associated with increased mortality with an OR of 6.25 (95% CI 1.66–23.5).

Our study is limited by its retrospective nature and small sample size. Despite the fact that our results are largely consistent with other published data, our study has introduced additional, earlier prognostication markers, allowing physicians to escalate treatment in a timely manner. Our findings warrant further investigation in a larger cohort prior to recommendation for wider usage.

## Conclusion

Mortality in patients who develop ACLF is high and there are no proven ways of accurately predicting the outcomes in such patients. Our study has demonstrated that minute changes in the MELD score at 2 weeks can provide early and useful prognostic information. The score is easy to calculate and uses variables that are routinely measured. Any improvement in the MELD score over 2 weeks suggests a good outcome. Conversely, a lack of improvement in the MELD score portends a poor outcome. The presence of cirrhosis does not appear to affect mortality in ACLF.

*Conflict of interest statement:* none declared.
